# High Seroprevalence of Hepatitis E Virus Infection among East Balkan Swine (*Sus scrofa*) in Bulgaria: Preliminary Results

**DOI:** 10.3390/pathogens9110911

**Published:** 2020-11-03

**Authors:** Ilia Tsachev, Magdalena Baymakova, Roman Pepovich, Nadezhda Palova, Plamen Marutsov, Krasimira Gospodinova, Todor Kundurzhiev, Massimo Ciccozzi

**Affiliations:** 1 Department of Microbiology, Infectious and Parasitic Diseases, Faculty of Veterinary Medicine, Trakia University, 6000 Stara Zagora, Bulgaria; ilia_tsachev@abv.bg (I.T.); plamen_marutsov@yahoo.com (P.M.); krasijel@abv.bg (K.G.); 2 Department of Infectious Diseases, Military Medical Academy, 1606 Sofia, Bulgaria; 3 Department of Infectious Pathology, Hygiene, Technology and Control of Foods from Animal Origin, Faculty of Veterinary Medicine, University of Forestry, 1797 Sofia, Bulgaria; rpepovich@abv.bg; 4 Scientific Center of Agriculture, 8300 Sredets, Bulgaria; aes_sredets@abv.bg; 5 Department of Occupational Medicine, Faculty of Public Health, Medical University, 1527 Sofia, Bulgaria; tgk_70@abv.bg; 6 Unit of Medical Statistics and Molecular Epidemiology, Universita Campus Bio-Medico di Roma, 00128 Rome, Italy; m.ciccozzi@unicampus.it

**Keywords:** Bulgaria, East Balkan swine, hepatitis E virus, seroprevalence, *Sus scrofa*

## Abstract

The East Balkan swine (*Sus scrofa*) is the only aboriginal pig breed in Bulgaria, and it is indigenous to the eastern part of the country. The aim of the present study was to investigate East Balkan swine (EBS) in Bulgaria for serological evidence of hepatitis E virus (HEV). Sera from 171 swine from two parts of the country (northeastern and southeastern) were tested for anti-HEV IgG antibodies. The overall HEV seroprevalence was 82.5% (141/171), and for weaners it was 77.2% (44/57), for fattening pigs 79.0% (45/57), and for adults 91.2% (52/57). HEV positivity was higher in fattening pigs and adults compared to weaners: OR = 1.108 (95% CI: 0.456–2.692) and OR = 3.073 (95% CI: 1.016–9.294), respectively. This study provides the first evidence of exposure to HEV in EBS from Bulgaria.

## 1. Introduction

**Figure 1:**
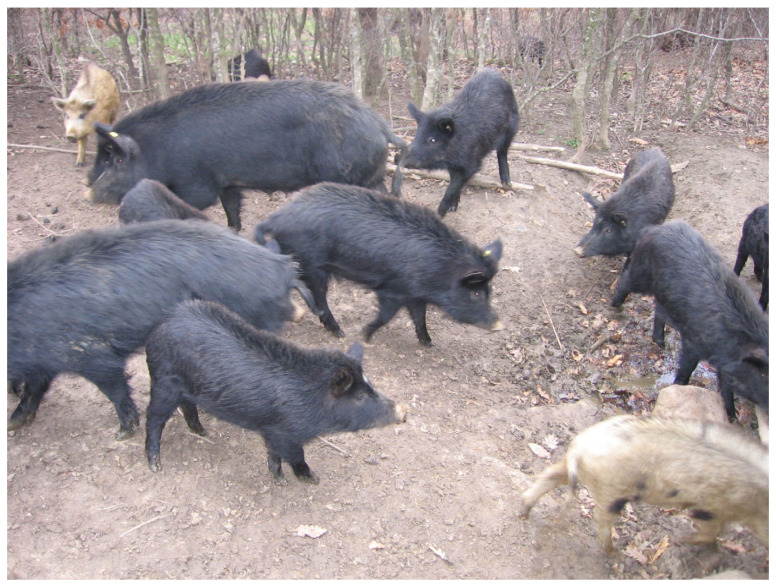
Anthropometric view of East Balkan swine (*Sus scrofa*).

The East Balkan swine (*Sus scrofa*) is the only aboriginal pig breed in Bulgaria. The East Balkan swine (EBS) is very healthy and resistant under adverse conditions. They are well-adapted to the continental climate and reared in wooded areas as well as on pastures. They have been reared outdoors for centuries in small (20–30 individuals) or in large groups (200–300 swine) [1]. The body is of short to medium length with a well-developed chest (Figure 1). The thoracic part is better developed than the croup. The hair color, bristles and hooves are usually black. The breed inhabits forests in eastern parts of the country. Nowadays, the population size of the breed is approximately 1500 sows [1].

In recent years, hepatitis E virus (HEV) has been increasingly common in humans and animals. HEV is a single-stranded positive-sense RNA virus [2,3]. HEV is classified into the *Hepeviridae* family, divided into two genera: *Ortohepevirus* and *Piscihepevirus* [2]. *Ortohepevirus* includes four species: *Orthohepevirus A*, *Orthohepevirus B*, *Orthohepevirus C* and *Orthohepevirus D* [2,4]. The genus *Piscihepevirus* has only one species—*Piscihepevirus A*, and one genotype—cutthroat trout HEV [2].

In Bulgaria, the first preliminary data for swine HEV seroprevalence were reported in 2018, and they show an overall seroprevalence of anti-HEV antibodies of 40% (34 positive samples out of all 85 sera) [5]. In 2019, a detailed seroprevalence study of HEV infection in pigs from southern Bulgaria documented an overall HEV seroprevalence of 60.3% (217 of all 360 tested sera) [6]. Takova et al., reported that 4 of the 32 meat juice samples from Bulgarian wild boar were positive for anti-HEV IgG antibodies [7]. The study of these authors had several important limitations: the research included a relatively small number of wild boars (*n* = 32), and information regarding their age and sex were not recorded.

However, there is no information about HEV infection in EBS from Bulgaria. Thus, the aim of the present study was to investigate the seroprevalence of HEV infection in East Balkan swine (*Sus scrofa*) in two parts of the country (northeastern and southeastern).

## 2. Materials and Methods

The study was approved by the Local Ethics Committee in Animal Experimentation and Animal Welfare at Trakia University, Stara Zagora, Bulgaria (Ethics Committee on Faculty of Veterinary Medicine at Trakia University, 6000 Stara Zagora, Protocol № 01/12 May 2017), and was conducted according to the ethical principles of animal experimentation, adopted by the Bulgarian Ministry of Agriculture, Food and Forestry.

**Figure 2:**
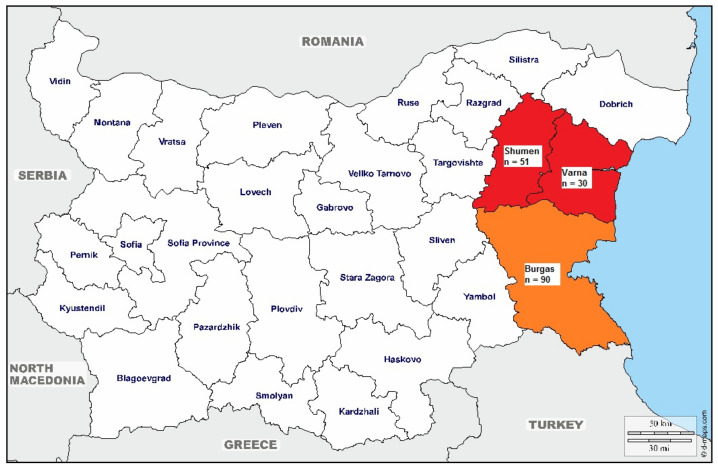
Geographic distribution of number of tested swine from the only aboriginal pig breed in Bulgaria—East Balkan swine (*Sus scrofa*). East Balkan swine (EBS) from the northeastern region are marked with red, and EBS from the southeastern region are marked with orange.

One hundred and seventy-one serum samples were collected from EBS (*n* = 171) and were enrolled from three districts in two parts of the country–the northeastern region (Varna and Shumen districts) and the southeastern region (Burgas district) (Figure 2). The samples were collected from January 2018 to December 2019. Animals were classified as weaners (between 30 and 100 days old), fattening pigs (between 101 and 160 days old) and adults (over 365 days old). The serum samples were collected from large herds (>150 individuals). An equal number of samples (*n* = 57) was taken from each age group. EBS showed no clinical signs at the sampling time point, and sex had not been recorded. The swine selection in age groups, respectively, in regions was conducted randomly. The geographic distribution of the investigated EBS was among mountains and hilly areas in eastern Bulgaria (approximately 26°40′ E and 27°84′ E longitude; 42°10′ N and 43°36′ N latitude), and the climate is continental (mean annual temperature: 13–15 °C, precipitations of about 650 mm/m^2^).

Swine blood samples (up to 5 mL per individual) were taken by puncture of the *sinus ophthalmicus*. Blood collection tubes without anticoagulant were kept at room temperature (20 °C) until visible clot retraction, centrifuged at 1500× *g* for ten minutes, and the serum was separated and kept at −20 °C until processing.

Serum samples were tested for HEV antibodies in the Laboratory of Infectious Diseases, Faculty of Veterinary Medicine, Trakia University, Stara Zagora, Bulgaria. A commercial enzyme-linked immunosorbent assay (ELISA, PrioCHECK HEV Ab porcine, Mikrogen GmbH, Neuried, Germany) was used. The PrioCHECK HEV Ab porcine is a diagnostic test for the detection of HEV-specific antibodies in porcine serum and meat juice samples. Microtiter plate is coated with recombinant HEV antigen of the open reading frame 2 (ORF2) and ORF3 of genotypes HEV-1 and HEV-3. The test has 91.0% sensitivity and 94.1% specificity. The cut-off value was calculated according to the manufacturer’s instructions. The cut-off is calculated as a mean optical density 450 (OD_450_) of the cut-off control multiplied with 1.2. Results obtained above or equal to the cut-off are considered positive. Results between the OD_450_ of the cut-off control and the cut-off are doubtful. Results obtained below the cut-off are negative.

HEV positive results among different swine age groups and regions were compared by the chi-square test. Binary logistic regression was used to evaluate the risk of positive results according to age group and according to regions. Statistical analysis was performed by Excel 2007 (Microsoft, Redmond, Washington, WA, USA) and SPSS Statistics 19.0 (IBM Corp., Armonk, New York, NY, USA). A *p*-value < 0.05 was considered statistically significant.

## 3. Results

Positive results for anti-HEV IgG were detected in 141 (82.5%) of all 171 tested sera (Table 1). The overall seropositivity in weaners, fattening pigs and adults was 77.2% (95% CI: 71.7–83.0), 79.0% (95% CI: 75.7–82.4), and 91.2% (95% CI: 82.9–98.9), respectively. The highest HEV seropositivity in the weaner age group was found in the northeastern region (22/27, 81.5%). The lowest HEV seropositivity in the fattening pig age group was estimated in the southeastern region (23/30, 76.7%). In the adult age group, the highest HEV seropositivity was observed in the southeastern region (29/30, 96.7%). The overall prevalence of anti-HEV antibodies in each region was as follows: 82.7% (67/81) in the northeastern region and 82.2% (74/90) in the southeastern region. Based on age groups and different regions, the chi-square test showed differences in HEV seropositivity between the age groups and regions (Table 1). Four percent of all samples were assessed as doubtful.

To estimate the risk for HEV seropositivity, the odds ratio (OR) in different age groups and regions was performed by binary logistic regression. The OR of anti-HEV antibody occurrence in fattening pigs and adults was determined in comparison to the weaner group (Table 2). The OR of anti-HEV antibody occurrence in the northeastern region was determined in comparison to the southeastern region (Table 3). We found that the odds of HEV infection were 1.108 times higher in fattening pigs and 3.073 times higher in adults than in the those in the weaner group. Furthermore, we found that the odds of HEV infection were 1.035 times higher in the northeastern region than in the southeastern region.

## 4. Discussion

EBS have been reared outdoors for centuries in small or in large groups, and, consequently, hybridization between EBS and Bulgarian wild boars is very likely to have occurred. The EBS is morphologically similar to the wild boar [1]. Hirata et al., reported an analysis of mitochondrial DNA (mtDNA) of EBS in Bulgaria [8]. These authors found that the median-joining network based on the mtDNA control region showed that the EBS and wild boar in Bulgaria comprised mainly two major mtDNA clades: European clade E1 (61.3%) and Asian clade A (38.7%). Scientific evidence of hybridization between domestic pigs and wild boars has been reported from Greece [9], Croatia [10], Italy, Slovenia and other Western Balkan regions [11], Belgium and Luxembourg [12], and Great Britain [13]. Georgiev and Benkov found that EBS crossed with imported pig breeds (Coloured German Swine, Mangalica, Berkshire, and Bulgarian White) [14,15]. Regarding the EBS population in the middle of the 20th century, they were the most common pig breed in Bulgaria. However, for many reasons, the number of EBS decreased in the following decades, and in current day, there remain approximately only 1500 sows [1]. The review of the scientific literature and historical data on EBS shows us that, first, it is a breed located between wild boars and domestic pigs, and, second, these swine are an endangered species.

As EBS is an aboriginal breed, we decided to compare HEV seropositivity among EBS to HEV prevalence in wild boars and domestic pigs, respectively. Low HEV seropositivity among wild boars was observed in countries of the Balkan Peninsula. Albayrak et al., reported that out of 93 serum samples examined, 93 (100.0%) were negative for HEV [16]. Porea et al., found overall prevalence of 9.61% (5/52) for anti-HEV antibodies among wild boars from Eastern Romania [17]. Zele et al. reported HEV seroprevalence 30.21% (87/288) of wild boar serum samples from Slovenia [18]. Jemersic et al. found 31.10% (311/1000; 95% CI: 28.31–34.04) HEV-positive wild boars in 6 of the 16 counties of Croatia [19]. Moderate and high HEV seropositivity among domestic pigs was observed in Southeastern Europe [20]. The estimated anti-HEV positivity in Croatia was 32.9% (469/1424) [19], in Serbia it was 34.6% (109/315) [21], in Romania 42.7% (65/145) and 49.3% (34/69) [22,23], and in Greece 80% (76/96) [24].

The reported data of studies from Balkan Peninsula countries showed considerably large variations in the established results. This variability could be influenced by diagnostics tests, study design and the tested population. Our results from the present study showed high HEV seropositivity among EBS. The reasons for this could be different. On the one hand, this breed lives in herds and in wooded areas, and has contact with both wild boars and domestic pigs. Consequently, there is potentially HEV transmission from wild boars to EBS and from domestic pigs to EBS. We supposed that EBS are more likely to become infected with HEV from domestic pigs. HEV seropositivity among domestic pigs in those districts is high, around 77.14–80.0% (slaughter-aged pigs, 6 months old) [7]. The HEV seroprevalence in wild boars in those districts has not yet been studied. Consequently, we cannot state what exactly the role of wild boars is in the process of HEV infecting of EBS. On the other hand, the diagnostic test (ELISA, PrioCHECK HEV Ab porcine, Mikrogen GmbH, Neuried, Germany) could have an influence on positive results. In our opinion, however, this is unlikely because among other swine from Italy and Germany, this test showed low HEV seropositivity of 4.9% (29/594; 95% CI: 3.3–6.9) and 11.5% (12/104), respectively [25,26].

Regarding the dynamics of HEV seroprevalence among different age groups, it was observed that positivity was lowest among weaners and highest among adults. The results of the present study showed an age-dependent seroprevalence among adults (OR = 3.073; *p* < 0.05). A similar relationship was observed in other studies from France and Switzerland [27,28]. 

This study has some limitations that need to be addressed. Since only the ELISA test was used for the detection of HEV antibodies without confirmatory testing, the results and the conclusions should be interpreted with caution. Moreover, the study included a relatively small number of pigs. Despite these limitations, this is the first study on anti-HEV prevalence among EBS in Bulgaria, which gives new insights for this infection in our country.

In conclusion, we present the first serological evidence of HEV infection among the only aboriginal pig breed in Bulgaria—East Balkan swine (*Sus scrofa*). The results of the present study showed high HEV seropositivity among EBS. This is important for public health because the potential transmission of HEV infection from swine to humans is well known. Therefore, attempts should be made to molecularly detect the virus genome in EBS and to characterize its nucleotide sequence. This is an important aim for future studies of HEV infection among EBS from Bulgaria.

## Figures and Tables

**Table 1:** Seroprevalence of hepatitis E virus (HEV) infection by age groups in East Balkan swine (*Sus scrofa*) from Bulgaria.

Age Groups	Investigated Swine, *n*	HEV Positive, *n* (%)	Chi-Square	df	*p*-Value
**Northeastern Region**					
Weaners	27	22 (81.5)	0.173	2	0.917
Fattening pigs	27	22 (81.5)			
Adults	27	23 (85.2)			
**Southeastern Region**					
Weaners	30	22 (73.3)	6.537	2	0.038
Fattening pigs	30	23 (76.7)			
Adults	30	29 (96.7)			

**Note**: HEV = hepatitis E virus; df = degrees of freedom.

**Table 2:** Logistic regression showing the relationship between HEV-positive East Balkan swine (*Sus scrofa*) and age group.

Age Group	Investigated Swine, *n*	HEV Positive, *n* (%)	PE	SE	*p*-Value	OR	95% CI
Weaners	57	44 (77.2)	NA	NA	NA	1.000	NA
Fattening pigs	57	45 (78.9)	0.103	0.435	0.821	1.108	0.456–2.692
Adults	57	52 (91.2)	1.123	0.565	0.047	3.073	1.016–9.294

**Note**: PE = parameter estimate; SE = standard error; OR = odds ratio; CI = confidence interval; NA = not applicable.

**Table 3:** Logistic regression showing the relationship between HEV-positive EBS and geographic distribution.

Region	Investigated Swine, *n*	HEV Positive, *n* (%)	PE	SE	*p*-Value	OR	95% CI
Southeastern	90	74 (82.2)	NA	NA	NA	1.000	NA
Northeastern	81	67 (82.7)	0.034	0.403	0.932	1.035	0.470–2.279
